# 3-Phenyl­coumarin

**DOI:** 10.1107/S1600536812034277

**Published:** 2012-08-04

**Authors:** Maria J. Matos, Lourdes Santana, Eugenio Uriarte

**Affiliations:** a Department of Organic Chemistry, University of Santiago de Compostela, Santiago de Compostela, Spain

## Abstract

In the title compound, C_15_H_10_O_2_, a 3-phenyl derivative of the coumarin (also known as 2*H*-chromen-2-one or 2*H*-1-benzopyran-2-one) scaffold, the C_p_—C_p_—C_c_—C_c_ torsion angle between the coumarin (c) ring system and the phenyl (p) ring is −47.6 (2)°.

## Related literature

For the synthesis of the title compound, see: Matos, Santana *et al.. et al.* (2011 ▶); Matos, Terán *et al.. et al.* (2011 ▶). For examples of biological activity of coumarin derivatives, see: Borges *et al.* (2009 ▶); Matos *et al.* (2009 ▶, 2010 ▶); Matos, Santana *et al.. et al.* (2011 ▶); Matos, Terán *et al.. et al.* (2011 ▶); Viña, Matos, Ferino *et al.* (2012 ▶); Viña, Matos Yáñez *et al.* (2012 ▶).
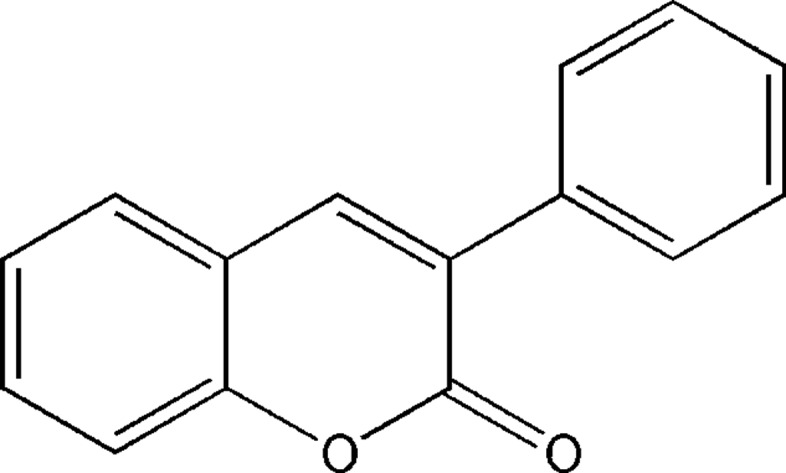


## Experimental

### 

#### Crystal data

C_15_H_10_O_2_
*M* *_r_* = 222.23
Monoclinic, 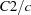

*a* = 18.469 (4) Å
*b* = 5.9596 (12) Å
*c* = 19.274 (4) Å
β = 99.079 (3)°
*V* = 2094.9 (7) Å^3^
*Z* = 8
Mo *K*α radiation
μ = 0.09 mm^−1^
*T* = 100 K
0.27 × 0.22 × 0.09 mm

#### Data collection

Bruker SMART CCD 1000 diffractometer
Absorption correction: multi-scan (*SADABS*; Bruker, 2002 ▶) *T*_min_ = 0.876, *T*_max_ = 1
9021 measured reflections
1924 independent reflections
1492 reflections with *I* > 2σ(*I*)
*R*_int_ = 0.042

#### Refinement

*R*[*F* ^2^ > 2σ(*F* ^2^)] = 0.040
*wR*(*F* ^2^) = 0.104
*S* = 1.03
1924 reflections
154 parameters
H-atom parameters constrained
Δρ_max_ = 0.21 e Å^−3^
Δρ_min_ = −0.26 e Å^−3^

### 

Data collection: *SMART* (Bruker, 2002 ▶); cell refinement: *SAINT* (Bruker, 2002 ▶); data reduction: *SAINT*; program(s) used to solve structure: *SIR97* (Altomare *et al.*, 1999 ▶); program(s) used to refine structure: *SHELXL97* (Sheldrick, 2008 ▶); molecular graphics: *ORTEP-3 for Windows* (Farrugia, 1997 ▶); software used to prepare material for publication: *WinGX* (Farrugia, 1999 ▶).

## Supplementary Material

Crystal structure: contains datablock(s) global, I. DOI: 10.1107/S1600536812034277/zj2091sup1.cif

Structure factors: contains datablock(s) I. DOI: 10.1107/S1600536812034277/zj2091Isup2.hkl

Supplementary material file. DOI: 10.1107/S1600536812034277/zj2091Isup3.cml

Additional supplementary materials:  crystallographic information; 3D view; checkCIF report

## Figures and Tables

**Fig. 1.**
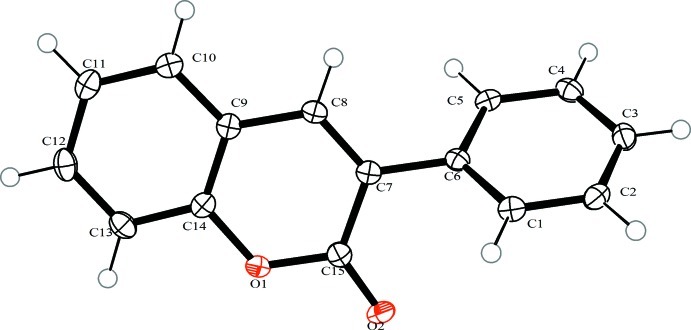
The molecular structure of the title compound with the atom-numbering scheme. Displacement ellipsoids are drawn at the 50% probability level.

**Fig. 2.**
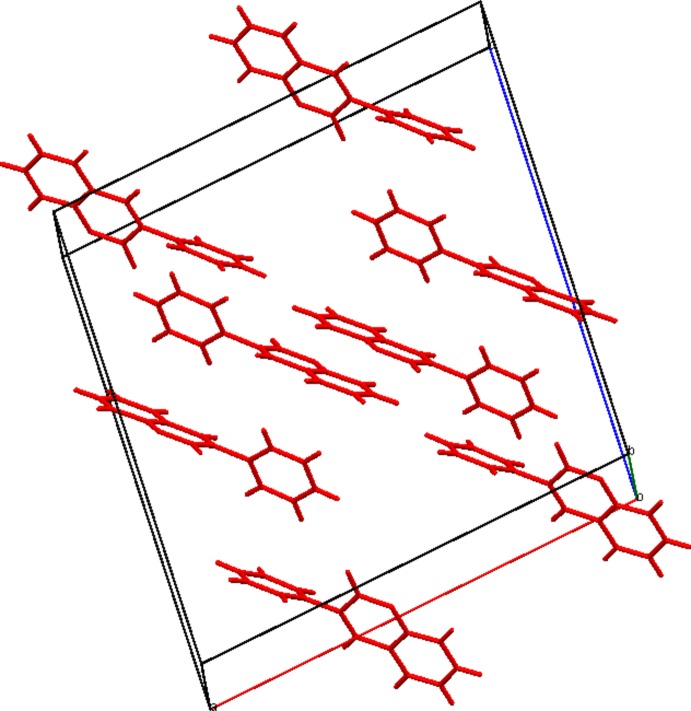
A packing diagram of the title structure viewed along the b axis.

**Fig. 3.**
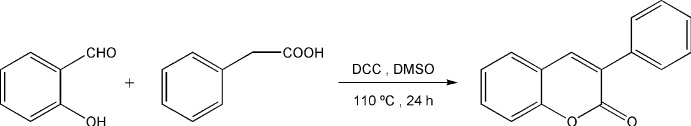
The formation of the title compound.

## supplementary crystallographic information

### Comment

Coumarin derivatives are very interesting molecules due to the biological properties that they may display (Borges *et al.* 2009; Matos *et al.* 2009, 2010; Matos, Santana *et al.*., 2011; Matos, Terán *et al.*., 2011). The title structure is a 3-phenyl coumarin derivative that posses one aromatic ring linked at that position. Therefore, the X-ray analysis of this compound (figure 1) aims to contribute to the elucidation of structural requirements needed to understand the partial planarity of the compound (coumarin nucleus) and the torsion of the 3-phenyl ring. From the single-crystal diffraction measurements one can conclude that the experimental bond lengths are within normal values with the average the molecule bond lengths. The planarity of the coumarin moiety is also evident by the torsion angles values between their carbons. Also, the angle C5—C6—C7—C8 is from -47.6 (2)°, typical of the torsion permitted by the rotation of the 3-phenyl ring. Packing diagram of the structure allows the interpretation of the spatial orientation of the molecules (figure 2).

### Experimental

3-Phenylcoumarin was prepared according to the protocol described by Matos, Santana *et al.*. *et al.* (2011) and Matos, Terán *et al.*. *et al.* (2011). Perkin reaction gave the desired coumarin derivative. A solution of 2-hydroxybenzaldehyde (0.9 g, 7.37 mmol) and the phenylacetic acid (1.25 g, 9.21 mmol) in dimethyl sulfoxide (15 ml) was prepared. Dicyclohexylcarbodiimide (DCC, 2.37 g, 11.50 mmol) was added, and the mixture was heated at 110 °C for 24 h. Ice (100 ml) and acetic acid (10 ml) were added to the reaction mixture. After keeping it at room temperature for 2 h, the mixture was extracted with ether (3 *x* 25 ml). The organic layer was extracted with sodium bicarbonate solution (50 ml, 5%) and then water (20 ml). The solvent was evaporated under vacuum, and the dry residue was purified by flash chromatography (hexane/ethyl acetate 9:1). A white solid was obtained in a yield of 67% (1.1 g). Suitable crystals for X-ray studies were grown from slow evaporation from acetone/ethanol: Mp. 131–132 °C; ^1^H NMR (300 MHz, CDCl_3_): *δ* 7.34–7.54 (5*H*, *m*, 6-H, 8-H, 9-H, 11-H, 13-H), 7.56–7.66 (2*H*, *m*, 10-H, 12-H), 7.72–7.80 (2*H*, *m*, 5-H, 7-H), 7.90 (1*H*, *s*, 4-H); ^13^C NMR (75.47 MHz, CDCl_3_): *δ* 116.5, 119.7, 124.5, 127.9, 128.4, 128.5, 128.5, 128.9, 131.4, 134.7, 139.9, 153.5, 160.6; DEPT: 116.5, 124.5, 127.9, 128.4, 128.5, 128.5, 131.4, 139.9; MS *m/z* 223 ([*M* + 1]^+^, 16), 222 (*M*^+^, 100). Anal. Calcd for C_15_H_10_O_2_: C, 81.07; H, 4.54. Found: C, 81.02; H, 4.52.

### Refinement

Refinement of *F*^2^ against ALL reflections. The weighted *R*-factor *wR* and goodness of fit *S* are based on *F*^2^, conventional *R*-factors *R* are based on *F*, with *F* set to zero for negative *F*2. The threshold expression of *F*^2^ > 2σ(*F*^2^) is used only for calculating *R*- factors(gt) *etc*. and is not relevant to the choice of reflections for refinement. *R*-factors based on *F*2 are statistically about twice as large as those based on *F*, and *R*- factors based on ALL data will be even larger.

### Crystal data

**Table:**

C_15_H_10_O_2_	*F*(000) = 928
*M**_r_* = 222.23	F(000) = 928
Monoclinic, *C*2/*c*	*D*_x_ = 1.409 Mg m^−^^3^
Hall symbol: -C 2yc	Mo *K*α radiation, λ = 0.7107 Å
*a* = 18.469 (4) Å	Cell parameters from 1813 reflections
*b* = 5.9596 (12) Å	θ = 2.8–26.2°
*c* = 19.274 (4) Å	µ = 0.09 mm^−^^1^
β = 99.079 (3)°	*T* = 100 K
*V* = 2094.9 (7) Å^3^	Prism, colourless
*Z* = 8	0.27 × 0.22 × 0.09 mm

### Data collection

**Table:**

Bruker SMART CCD 1000 diffractometer	1924 independent reflections
Radiation source: fine-focus sealed tube	1492 reflections with *I* > 2σ(*I*)
Graphite monochromator	*R*_int_ = 0.042
ω scans	θ_max_ = 25.4°, θ_min_ = 2.1°
Absorption correction: multi-scan (*SADABS*; Bruker, 2002)	*h* = −22→21
*T*_min_ = 0.876, *T*_max_ = 1	*k* = 0→7
9021 measured reflections	*l* = 0→23

### Refinement

**Table:**

Refinement on *F*^2^	Primary atom site location: structure-invariant direct methods
Least-squares matrix: full	Secondary atom site location: difference Fourier map
*R*[*F*^2^ > 2σ(*F*^2^)] = 0.040	Hydrogen site location: inferred from neighbouring sites
*wR*(*F*^2^) = 0.104	H-atom parameters constrained
*S* = 1.03	*w* = 1/[σ^2^(*F*_o_^2^) + (0.0528*P*)^2^ + 1.3579*P*] where *P* = (*F*_o_^2^ + 2*F*_c_^2^)/3
1924 reflections	(Δ/σ)_max_ = 0.001
154 parameters	Δρ_max_ = 0.21 e Å^−^^3^
0 restraints	Δρ_min_ = −0.26 e Å^−^^3^

### Special details

**Table:**

Geometry. All s.u.'s (except the s.u. in the dihedral angle between two l.s. planes) are estimated using the full covariance matrix. The cell s.u.'s are taken into account individually in the estimation of s.u.'s in distances, angles and torsion angles; correlations between s.u.'s in cell parameters are only used when they are defined by crystal symmetry. An approximate (isotropic) treatment of cell s.u.'s is used for estimating s.u.'s involving l.s. planes.
Refinement. Refinement of *F*^2^ against ALL reflections. The weighted *R*-factor *wR* and goodness of fit *S* are based on *F*^2^, conventional *R*-factors *R* are based on *F*, with *F* set to zero for negative *F*^2^. The threshold expression of *F*^2^ > 2σ(*F*^2^) is used only for calculating *R*-factors(gt) *etc*. and is not relevant to the choice of reflections for refinement. *R*-factors based on *F*^2^ are statistically about twice as large as those based on *F*, and *R*- factors based on ALL data will be even larger.

### Fractional atomic coordinates and isotropic or equivalent isotropic displacement parameters (Å^2^)

**Table:**

	*x*	*y*	*z*	*U*_iso_*/*U*_eq_	
C1	0.21370 (8)	0.2410 (3)	0.36971 (8)	0.0168 (4)	
H1	0.219	0.3712	0.3985	0.02*	
C2	0.15672 (9)	0.2280 (3)	0.31395 (8)	0.0184 (4)	
H2	0.1223	0.3472	0.3054	0.022*	
C3	0.14979 (9)	0.0409 (3)	0.27041 (8)	0.0192 (4)	
H3	0.1115	0.0337	0.2313	0.023*	
C4	0.19876 (9)	−0.1348 (3)	0.28427 (8)	0.0188 (4)	
H4	0.1939	−0.263	0.2546	0.023*	
C5	0.25492 (9)	−0.1254 (3)	0.34124 (8)	0.0166 (4)	
H5	0.2876	−0.2485	0.3511	0.02*	
C6	0.26348 (8)	0.0646 (3)	0.38409 (8)	0.0145 (4)	
C7	0.32522 (8)	0.0774 (3)	0.44333 (8)	0.0146 (3)	
C8	0.34288 (8)	−0.0903 (3)	0.49004 (8)	0.0145 (3)	
H8	0.3139	−0.2228	0.4858	0.017*	
C9	0.40398 (8)	−0.0744 (3)	0.54588 (8)	0.0145 (4)	
C10	0.42315 (8)	−0.2408 (3)	0.59681 (8)	0.0168 (4)	
H10	0.3949	−0.3744	0.5955	0.02*	
C11	0.48286 (9)	−0.2116 (3)	0.64877 (8)	0.0201 (4)	
H11	0.4954	−0.3246	0.6834	0.024*	
C12	0.52497 (9)	−0.0168 (3)	0.65072 (8)	0.0206 (4)	
H12	0.5664	0.001	0.6863	0.025*	
C13	0.50708 (9)	0.1506 (3)	0.60137 (8)	0.0190 (4)	
H13	0.5357	0.2835	0.6026	0.023*	
C14	0.44646 (8)	0.1196 (3)	0.55016 (8)	0.0152 (4)	
C15	0.37106 (8)	0.2799 (3)	0.44882 (8)	0.0161 (4)	
O1	0.42953 (6)	0.29055 (18)	0.50224 (5)	0.0177 (3)	
O2	0.36238 (6)	0.43926 (18)	0.40948 (6)	0.0220 (3)	

### Atomic displacement parameters (Å^2^)

**Table:**

	*U*^11^	*U*^22^	*U*^33^	*U*^12^	*U*^13^	*U*^23^
C1	0.0191 (8)	0.0165 (8)	0.0160 (8)	0.0006 (7)	0.0062 (7)	−0.0010 (7)
C2	0.0167 (8)	0.0186 (9)	0.0206 (8)	0.0025 (7)	0.0050 (7)	0.0052 (7)
C3	0.0166 (8)	0.0250 (9)	0.0156 (8)	−0.0040 (7)	0.0013 (6)	0.0034 (7)
C4	0.0215 (9)	0.0188 (9)	0.0167 (8)	−0.0050 (7)	0.0051 (7)	−0.0029 (7)
C5	0.0188 (8)	0.0134 (8)	0.0182 (8)	0.0012 (7)	0.0050 (7)	0.0020 (6)
C6	0.0144 (8)	0.0156 (8)	0.0144 (8)	−0.0023 (6)	0.0054 (6)	0.0004 (6)
C7	0.0149 (8)	0.0147 (8)	0.0152 (8)	0.0012 (6)	0.0055 (6)	−0.0020 (6)
C8	0.0154 (8)	0.0132 (8)	0.0160 (8)	0.0001 (6)	0.0058 (6)	−0.0028 (6)
C9	0.0138 (8)	0.0167 (8)	0.0140 (8)	0.0030 (6)	0.0056 (6)	−0.0019 (6)
C10	0.0174 (8)	0.0166 (8)	0.0177 (8)	0.0012 (7)	0.0066 (7)	0.0003 (7)
C11	0.0216 (9)	0.0225 (9)	0.0163 (8)	0.0063 (7)	0.0037 (7)	0.0017 (7)
C12	0.0158 (8)	0.0283 (10)	0.0171 (8)	0.0035 (7)	0.0010 (6)	−0.0041 (7)
C13	0.0174 (8)	0.0201 (9)	0.0199 (9)	−0.0013 (7)	0.0046 (7)	−0.0057 (7)
C14	0.0168 (8)	0.0162 (8)	0.0136 (8)	0.0038 (7)	0.0057 (6)	−0.0005 (6)
C15	0.0154 (8)	0.0161 (8)	0.0171 (8)	0.0020 (7)	0.0038 (6)	−0.0025 (7)
O1	0.0202 (6)	0.0138 (6)	0.0185 (6)	−0.0026 (5)	0.0011 (5)	0.0000 (5)
O2	0.0239 (6)	0.0148 (6)	0.0265 (6)	−0.0003 (5)	0.0017 (5)	0.0053 (5)

### Geometric parameters (Å, º)

**Table:**

C1—C2	1.382 (2)	C8—H8	0.95
C1—C6	1.395 (2)	C9—C14	1.392 (2)
C1—H1	0.95	C9—C10	1.401 (2)
C2—C3	1.390 (2)	C10—C11	1.379 (2)
C2—H2	0.95	C10—H10	0.95
C3—C4	1.382 (2)	C11—C12	1.395 (2)
C3—H3	0.95	C11—H11	0.95
C4—C5	1.387 (2)	C12—C13	1.382 (2)
C4—H4	0.95	C12—H12	0.95
C5—C6	1.396 (2)	C13—C14	1.383 (2)
C5—H5	0.95	C13—H13	0.95
C6—C7	1.483 (2)	C14—O1	1.3776 (18)
C7—C8	1.350 (2)	C15—O2	1.2102 (19)
C7—C15	1.468 (2)	C15—O1	1.3709 (19)
C8—C9	1.434 (2)		
			
C2—C1—C6	120.61 (15)	C9—C8—H8	119
C2—C1—H1	119.7	C14—C9—C10	117.94 (14)
C6—C1—H1	119.7	C14—C9—C8	117.93 (14)
C1—C2—C3	120.03 (15)	C10—C9—C8	124.13 (15)
C1—C2—H2	120	C11—C10—C9	120.29 (15)
C3—C2—H2	120	C11—C10—H10	119.9
C4—C3—C2	119.76 (15)	C9—C10—H10	119.9
C4—C3—H3	120.1	C10—C11—C12	120.22 (15)
C2—C3—H3	120.1	C10—C11—H11	119.9
C3—C4—C5	120.48 (15)	C12—C11—H11	119.9
C3—C4—H4	119.8	C13—C12—C11	120.71 (15)
C5—C4—H4	119.8	C13—C12—H12	119.6
C4—C5—C6	120.11 (15)	C11—C12—H12	119.6
C4—C5—H5	119.9	C12—C13—C14	118.25 (15)
C6—C5—H5	119.9	C12—C13—H13	120.9
C1—C6—C5	118.96 (14)	C14—C13—H13	120.9
C1—C6—C7	121.09 (14)	O1—C14—C13	116.88 (14)
C5—C6—C7	119.94 (14)	O1—C14—C9	120.55 (14)
C8—C7—C15	118.97 (14)	C13—C14—C9	122.57 (14)
C8—C7—C6	123.41 (14)	O2—C15—O1	116.41 (14)
C15—C7—C6	117.56 (14)	O2—C15—C7	125.59 (15)
C7—C8—C9	122.02 (15)	O1—C15—C7	117.99 (14)
C7—C8—H8	119	C15—O1—C14	122.53 (12)
			
C6—C1—C2—C3	−1.8 (2)	C9—C10—C11—C12	−0.5 (2)
C1—C2—C3—C4	1.9 (2)	C10—C11—C12—C13	0.8 (2)
C2—C3—C4—C5	−0.2 (2)	C11—C12—C13—C14	0.0 (2)
C3—C4—C5—C6	−1.6 (2)	C12—C13—C14—O1	179.25 (13)
C2—C1—C6—C5	0.0 (2)	C12—C13—C14—C9	−1.2 (2)
C2—C1—C6—C7	179.41 (14)	C10—C9—C14—O1	−178.93 (13)
C4—C5—C6—C1	1.6 (2)	C8—C9—C14—O1	0.4 (2)
C4—C5—C6—C7	−177.73 (14)	C10—C9—C14—C13	1.5 (2)
C1—C6—C7—C8	133.05 (16)	C8—C9—C14—C13	−179.15 (13)
C5—C6—C7—C8	−47.6 (2)	C8—C7—C15—O2	178.09 (15)
C1—C6—C7—C15	−49.86 (19)	C6—C7—C15—O2	0.9 (2)
C5—C6—C7—C15	129.50 (15)	C8—C7—C15—O1	−0.9 (2)
C15—C7—C8—C9	1.5 (2)	C6—C7—C15—O1	−178.17 (12)
C6—C7—C8—C9	178.59 (14)	O2—C15—O1—C14	−179.01 (13)
C7—C8—C9—C14	−1.3 (2)	C7—C15—O1—C14	0.1 (2)
C7—C8—C9—C10	178.02 (14)	C13—C14—O1—C15	179.72 (13)
C14—C9—C10—C11	−0.6 (2)	C9—C14—O1—C15	0.1 (2)
C8—C9—C10—C11	−179.96 (14)		
